# Penalized negative binomial models for modeling an overdispersed count outcome with a high-dimensional predictor space: Application predicting micronuclei frequency

**DOI:** 10.1371/journal.pone.0209923

**Published:** 2019-01-08

**Authors:** Rebecca R. Lehman, Kellie J. Archer

**Affiliations:** 1 United Network for Organ Sharing, Richmond, VA, United States of America; 2 Division of Biostatistics, College of Public Health, The Ohio State University, Columbus, OH, United States of America; Universita degli Studi del Piemonte Orientale Amedeo Avogadro, ITALY

## Abstract

Chromosomal aberrations, such as micronuclei (MN), have served as biomarkers of genotoxic exposure and cancer risk. Guidelines for the process of scoring MN have been presented by the HUman MicroNucleus (HUMN) project. However, these guidelines were developed for assay performance but do not address how to statistically analyze the data generated by the assay. This has led to the application of various statistical methods that may render different interpretations and conclusions. By combining MN with data from other high-throughput genomic technologies such as gene expression microarray data, we may elucidate molecular features involved in micronucleation. Traditional methods that can model discrete (synonymously, count) data, such as MN frequency, require that the number of explanatory variables (*P*) is less than the number of samples (*N*). Due to this limitation, penalized models have been developed to enable model fitting for such over-parameterized datasets. Because penalized models in the discrete response setting are lacking, particularly when the count outcome is over-dispersed, herein we present our penalized negative binomial regression model that can be fit when *P* > *N*. Using simulation studies we demonstrate the performance of our method in comparison to commonly used penalized Poisson models when the outcome is over-dispersed and applied it to MN frequency and gene expression data collected as part of the Norwegian Mother and Child Cohort Study. Our countgmifs R package is available for download from the Comprehensive R Archive Network and can be applied to datasets having a discrete outcome that is either Poisson or negative binomial distributed and a high-dimensional covariate space.

## Introduction

**Fig 1:**
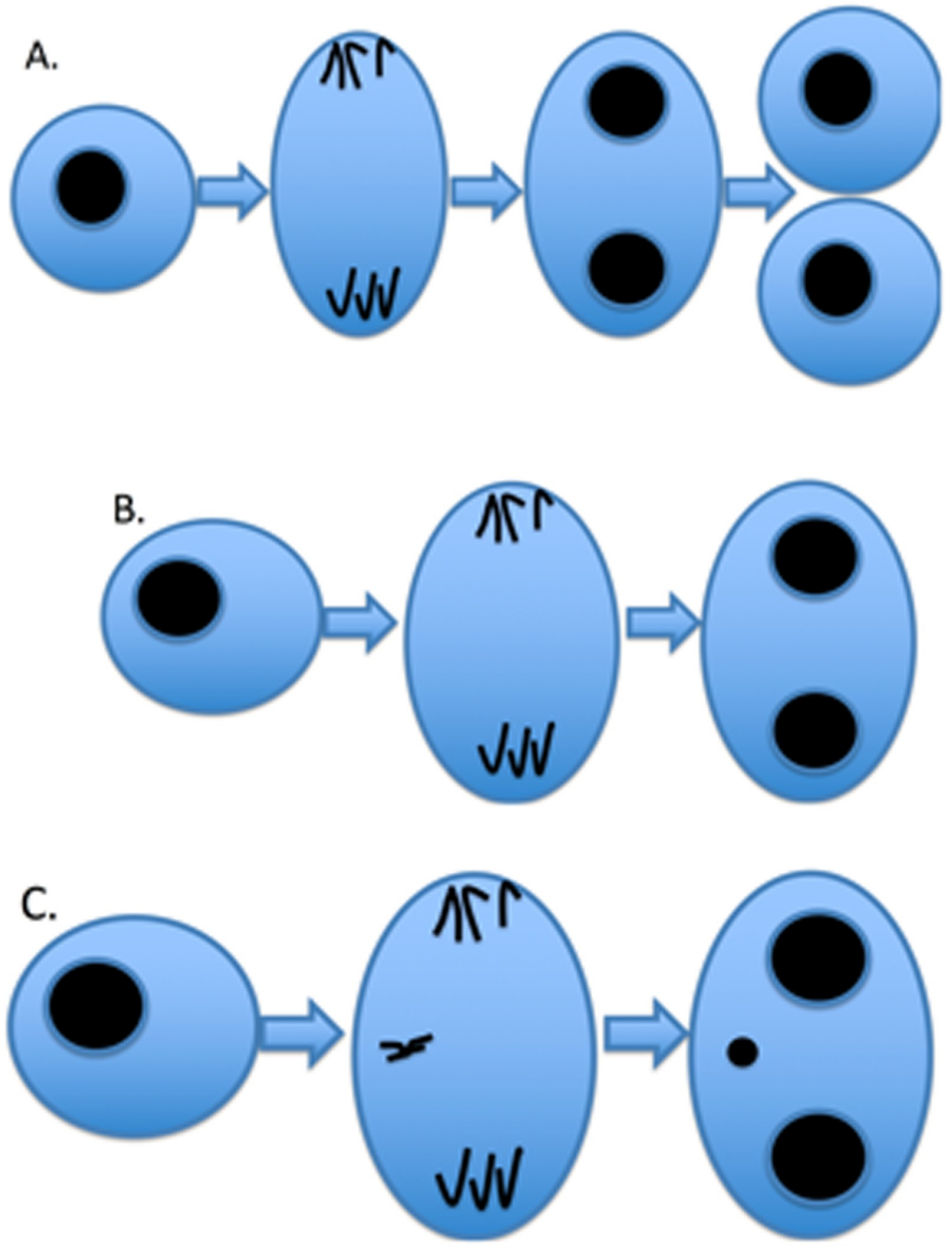
CBMN/Cytome assay. A: Typical process of cell division. B: Application of cytochalasin-B prevents cytokinesis to give rise to binucleated cells. C: Cell treated with cytochalasin-B that contains a whole chromosome lagging behind that does not attach to the mitotic spindle. The small extranuclear body in the binucleated cell is a MN.

More than 85% of all cancers are associated with acquired chromosomal or genetic alterations. Various cytogenetic endpoints have been used for cancer risk assessment, including structural chromosomal aberrations, aneuploidy, while sister chromatid exchanges have been useful biomarkers of exposure [1]. Micronuclei (MN) have also been used both for cancer risk assessment and to assess exposure to genotoxic agents. Micronuclei (MN) are formed in dividing cells from either whole chromosomes or chromosome fragments that lag behind [2], do not attach to the mitotic spindle prior to cytokinesis, so that after cell division, they appear to be small extranuclear bodies (Fig 1). The presence of MN, particularly at high frequencies, is taken to reflect chromosomal abnormalities. Various investigators have reported higher MN frequencies among people who were exposed to a toxic agent compared to controls not exposed [3–6]. Also, previous research has shown that higher MN frequencies are associated with a higher risk of cancer development [7, 8] and that MN frequencies are higher in subjects with cancer compared to those without cancer [9–14]. As a specific example, MN formation was increased due to genotoxic hepatocarcinogen exposure compared to non-genotoxic exposure [15], indicating MN formation is due to chromosomal damage. Thus, MN serve as an early marker for carcinogenesis. In fact, MN frequency has been used in biomonitoring [16, 17], occupational exposure [3, 4, 18–20], environmental exposure [5, 6, 21–24], and as previously mentioned, in cancer research studies [7, 9–14, 25–31]. MN frequency is also relevant in other developmental, age-related, degenerative diseases (e. g. Alzheimer’s disease, Parkinson’s disease) [6]. Therefore, because MN are objectively measured they are a useful biomarker for assessing both genotoxic exposure and cancer risk and may serve as an indicator of a pathogenic process.

The Cytokinesis block micronucleus assay (CBMN) assay is commonly used to score MN. Briefly, the CBMN assay uses cytochalasin-B, which stops cells from performing cytokinesis but does not stop nuclear division, giving rise to cells that are binucleated [2, 8]. Guidelines for scoring MNs have been established by the HUman MicroNucleus (HUMN) project to minimize inter-rater variation [6, 32]. MN frequency is generally reported as the number of binucleated cells containing at least one MN. Therefore, MN frequency is a discrete or count variable. Other unique nuclear anomalies detectable using the CBMN/cytome assay include nucleoplasmic bridges and nuclear buds, which seem to be caused by different mechanisms in comparison to MN [33]. In fact, buds, also called broken eggs, are considered to be a marker of gene amplification, being conjectured to arise due to errors in replication during the S phase of the cell cycle [32, 34]. Thus scoring each of these anomalies provides a comprehensive assessment of genotoxic exposure and genetic damage [35, 36].

**Fig 2:**
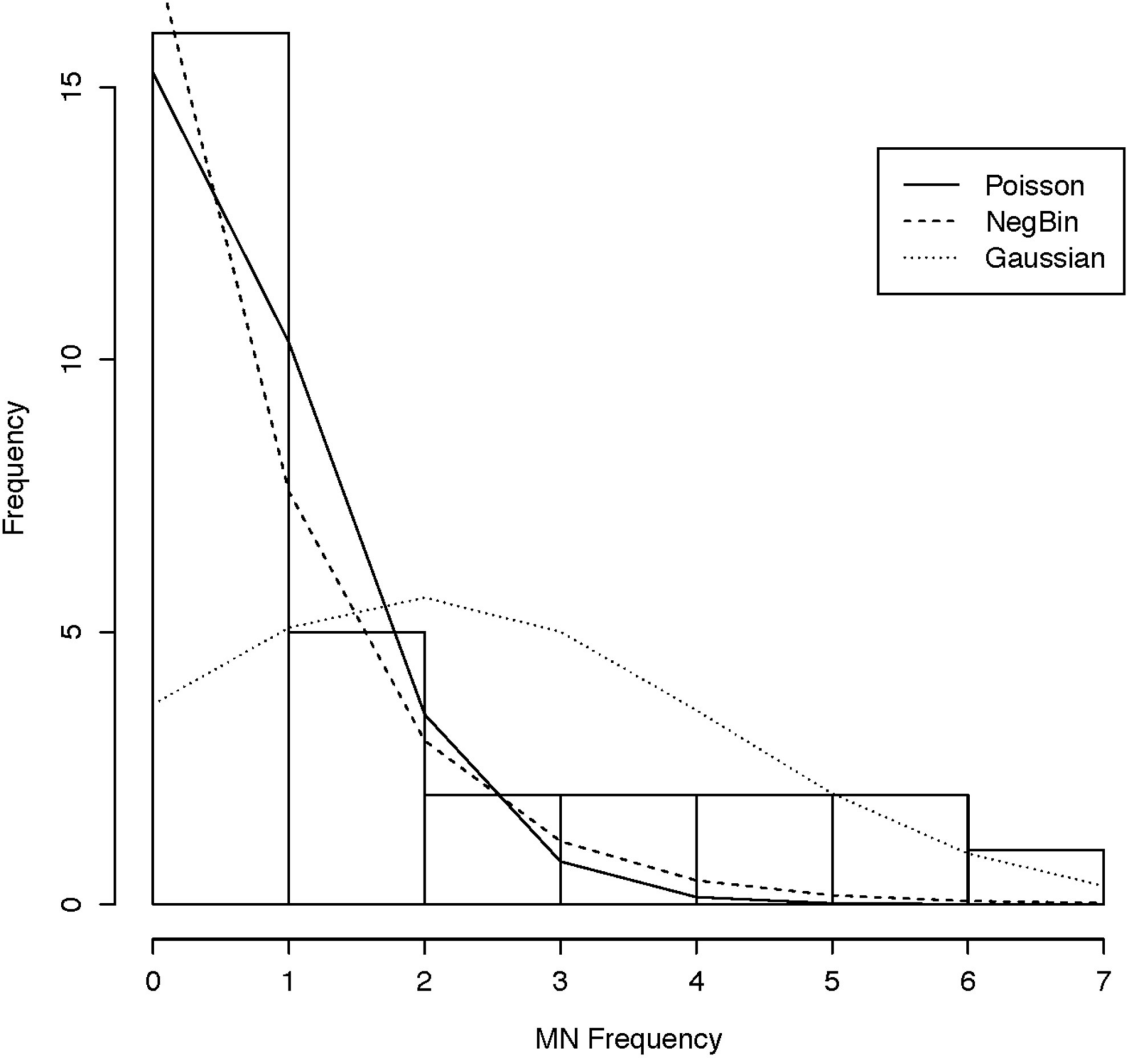
MN frequency distribution. Histogram of MN frequency from the cheek opposite an oral carcinoma with lines representing the Poisson (solid), Negative Binomial (dashed), and Gaussian (dotted) distributions. Data from Ramirez & Saldanha [39].

Although the HUMN project developed guidelines for assay performance, they have not addressed how to statistically analyze data generated by the assay. Ceppi et al [37] reviewed 63 studies that analyzed MN frequency in buccal cells with respect to their study design and analytical methods applied. Most frequently the studies involved two-group comparisons so that the t-test, the non-parametric Mann-Whitney U-test were most frequently applied (38.1%, 31.7% of studies, respectively). Although the non-parametric tests applied do not require an underlying distributional assumption, they are unable to adjust for confounding factors and often result in a loss of statistical power. While linear regression, ANOVA, and ANCOVA can be used to adjust for confounding variables and effect modifiers, problematically MN frequency rarely follows a Gaussian distribution (Fig 2). As mentioned, MN frequency is a discrete count outcome. Discrete probability distributions differ from those for continuous (takes on an infinite number of possible values), nominal (categorical variable), and ordinal (≥ 3-level categorical variable where the categories have a natural ordering imposed, such as small, medium, large) variables. When MN frequency is over-dispersed, that is, the distribution is not well-described by the mean and variance having the same parameter, the discrete negative binomial (NB) distribution fits better than the Poisson distribution, while the Gaussian distribution poorly fits the MN data (Fig 2). Over-dispersion may be due to the data including more zero count responses than expected, positive correlation between the count responses, or due to correlation between an explanatory variable and the error term [38]. The skewed distribution of MN frequency demonstrates excess zeros, and suggests that methods based on the Gaussian distribution may lead to inappropriate inferences. Unfortunately, authors have not routinely reported whether basic assumptions of the inferential tests applied were met [37].

Among the 63 studies reviewed [37], non-Gaussian multivariable models applied included logistic (*N* = 1), log-linear (*N* = 1), Poisson (*N* = 1), and NB regression (*N* = 4). The logistic and log-linear models only model categorical data (e.g., MN are present versus absent) whereas Poisson and NB directly model count data. Because MN frequency is generally reported as the number of binucleated cells containing at least one MN, accounting for the number of binucleated cells scored is important. Therefore, advantages of the Poisson and NB models include that they: (1) are well-suited for modeling skewed count distributions; (2) can adjust for confounding variables such as age, gender, smoking status [37]; and (3) can account for the total number of cells scored per patient, which may vary from sample to sample. For these reasons, Ceppi et al [37] recommended using NB or Poisson regression models when analyzing MN data.

In the last decade, high-throughput genomic platforms have been increasingly used to identify molecular features associated with disease status, exposure, or outcome. Recently, some studies have collected both MN frequency and gene expression data to confirm the genotoxicity of the exposure studied and to assess the molecular impact of exposure, respectively. Unfortunately, the association between gene expression and MN frequency was often not explored [15, 40, 41]. We know that many disease-causing mutations do not have complete penetrance, and penetrance is affected by environmental factors, age, epigenetics, and other genetic modifiers. Moreover, conditions with a complex inheritance pattern, such as cancer, are likely polygenic and have environmental contributions.

As previously mentioned, MN frequency is a discrete count outcome. Though Poisson and negative binomial regression can model a discrete response, they traditionally require that the number of covariates (*P*) is less than the number of samples (*N*) [38]. However, in high-throughput genomic datasets, *P* > *N*, often by several orders of magnitude. While methods such as principle components analysis have been used to reduce the total number of variables in the dataset, thus permitting model fitting, the resulting features are complicated linear combinations of genomic features, making downstream gene interpretations difficult. As an alternative, the least absolute shrinkage and selection operator (LASSO) [42, 43], also referred to as L1 penalized models, can be used to fit continuous [44, 45], binary [46, 47], and survival [48–51] models when *P* > *N*. Downstream gene interpretations are more straight-forward, because the original features are included in the model (though they are often first centered and scaled) [42, 43]. There are various computational approaches for estimating L1 penalized models including least angle regression (LARS) for the ordinary linear regression setting [52] and the predictor-corrector [53] and cyclical coordinate descent [54] algorithms for generalized linear models. Also, Hastie et al (2007) demonstrated that the solution path of the incremental forward stagewise (IFS) method is equivalent to the LASSO when the LASSO path is monotone for each coefficient and Efron et al (2004) described necessary and sufficient conditions for every coefficient path to be monotone. IFS was described in detail for ordinary linear regression but also for general convex loss functions, with logistic regression as a specific example [55]. For general convex loss functions, incremental updates are made to the coefficient having the smallest negative gradient of the log-likelihood at the current model estimates, hence the algorithm is called the generalized monotone incremental forward stagewise method. While the incremental nature of the algorithm naturally leads to longer computational time, the monotone paths are much smoother than those produced by other L1 algorithms which sometimes yield widely fluctuating paths, particularly when covariates are highly correlated. We have also recently extended the generalized monotone incremental forward stagewise (GMIFS) method [55] to high-dimensional ordinal response [56–61] and Poisson regression [62] settings. However, penalized methods have not been fully extended to discrete response setting when over-dispersion is present. We postulate that it is of interest to identify molecular features associated with micronucleation, as such features may elucidate important mechanisms in genotoxicity and carcinogenesis. Herein we describe a multivariable discrete response modeling method that can be applied to a high-dimensional covariate space when the count outcome is over-dispersed, such as the case for MN data.

## Materials and methods

### Poisson regression

When modeling the number of times an event occurs in either time or space, a generalized linear model (GLM) such as Poisson or negative binomial regression is commonly applied. Let *i* = 1, …, *N* be the number of observations, *y*_*i*_ represent a Poisson distributed random variable. Let the expected value of *y*_*i*_ be written as $E\left(y_{i}\right)=\mu_{i}$. Then the conditional probability *P*(*y*_*i*_|*μ*_*i*_) for each observation *i*, subsequently the likelihood *L*(*μ*|**y**) are represented by

$$\begin{array}{c} P\left(y_{i}|\mu_{i}\right)=\frac{{\text{e}}^{-\mu_{i}}\mu_{i}^{y_{i}}}{y_{i}!}\;\text{and}\;L\left(\mu |y\right)=\prod_{i=1}^{N}\frac{{\text{e}}^{-\mu_{i}}\mu_{i}^{y_{i}}}{y_{i}!}. \end{array}$$ (1)

Mathematically it is easier to maximize the log-likelihood which is given by

$$\begin{array}{c} \ell \left(\mu |y\right)=\sum_{i=1}^{N}\left(y_{i}\log\mu_{i}-\mu_{i}-\log\left(y_{i}!\right)\right). \end{array}$$ (2)

Thus, we are looking for the value of *μ* that maximizes the log-likelihood in Eq 2. When fitting a GLM, a non-linear transformation, or link function, of the mean response is applied, which is a linear function of the covariates [38]. The link function for a Poisson regression model is log(*μ*), thus, $\mu_{i}=\exp\left(\gamma_{0}+x_{i}^{⊤}\beta \right)$ where *γ*_0_ is the intercept and ***β*** is the vector of regression coefficients. Further, MN frequency is scored from a large number of binucleated cells, *c*. If the number of cells examined varies by subject, modeling MN frequency as a rate is more appropriate. We note that expressing a discrete response as a rate then transforming the rate to get it to adhere to a Gaussian distribution so that traditional linear models can be fit, does not properly account for the variation observed in the numerator and denominator terms. Therefore, Poisson and NB regression models that explicitly include the denominator term are more appropriate. The Poisson regression model for the expected count per unit of *c*_*i*_, where *c*_*i*_ is the number of cells scored in our application, is $E\left(y_{i}/c_{i}\right)=\mu_{i}=\exp\left(\gamma_{0}+x_{i}^{⊤}\beta \right)$ which is equivalent to

$$\begin{array}{c} \mu_{i}=\exp\left(\gamma_{0}+x_{i}^{⊤}\beta +\log\left(c_{i}\right)\right) \end{array}$$ (3)

so that log(*c*_*i*_) is an offset term. Traditionally count models are estimated by maximum likelihood or an iteratively re-weighted least squares algorithm [38]. For the rate-based model, the log-likelihood expressed with covariates is

$$\begin{array}{c} \ell \left(\mu |y\right)=\sum_{i=1}^{N}\left(y_{i}\left(\gamma_{0}+x_{i}^{⊤}\beta +\log\left(c_{i}\right)\right)-\exp\left(\gamma_{0}+x_{i}^{⊤}\beta +\log\left(c_{i}\right)\right)-\log\left(y_{i}!\right)\right). \end{array}$$ (4)

### Negative binomial regression

For the Poisson regression model, the distributional assumption is that the responses are from a Poisson distribution, which is a member of the exponential family. Members of the exponential family have a common characteristic that the variance of the response can be expressed as the product of a dispersion parameter, *ϕ*, and the variance function, Var(*μ*_*i*_). In other words, Var(*Y*_*i*_) = *ϕ*Var(*μ*_*i*_). For the Poisson distribution, the dispersion parameter is a fixed constant (*ϕ* = 1) and does not require estimation; therefore, Var(*μ*_*i*_) = *μ*.

Because the Poisson distribution has one parameter *μ* which represents both the mean and variance of the count outcome, the Poisson model is equi-dispersed. As previously mentioned, over-dispersion is present when the variance exceeds the mean, which is a common occurrence in observed data. If not properly accounted for, over-dispersion results in incorrect standard errors and thus potentially incorrect inferences. The Pearson dispersion can be examined as a means for checking for over-dispersion in a Poisson regression model. If the Pearson dispersion exceeds 1.0, over-dispersion may be present. Alternative models such as the NB model, zero-inflated models, truncated models, or quantile count models can be used, though NB regression was found to best handle over-dispersed discrete response data [63]. Extensive work has been done with the Poisson and negative binomial distributions in the traditional statistical setting. However, there are limited methods for analyzing a count outcome with a high-dimensional predictor space.

### Penalized Poisson regression

Development of high-dimensional methods for count response data have largely been restricted to the Poisson distribution [53, 54, 62, 64], especially for longitudinal settings [65–67]. The glmpath [53], glmnet [54], and nnlasso [64]R packages each provide an L_1_ regularization path algorithm that seek either a LASSO solution, which minimizes $-\ell \left(\beta ;y\right)+\lambda \sum_{p=1}^{P}\left|\beta_{p}\right|$, or an elastic net solution by estimating coefficients over a grid of λ values, where λ > 0 is the regularization parameter. Basically, glmpath starts with the smallest value of the regularization parameter, λ_*max*_, at which only the intercept is non-zero. Thereafter, a grid of λ values from λ_*max*_ to 0 is identified which allows other covariates to enter the model. The method uses the predictor-corrector algorithm to estimate the next value of λ that will change the current set of non-zero parameter estimates in the model (the active set) and then finds the solution to ***β*** at that value of λ. The glmnet algorithm also starts with the smallest value of the regularization parameter, λ_*max*_, at which only the intercept is non-zero but then uses a sequence of 100 λ values that are evenly spaced on the log-scale. It uses cyclical coordinate descent to solve the elastic net penalized model, which minimizes $-\ell \left(\beta ;y,\alpha \right)+\lambda (\sum_{p=1}^{P}(\frac{1}{2}\left(1-\alpha \right)\beta_{p}^{2}+\alpha |\beta_{p}\left|\right))$ where *α* is a fixed proportion representing a compromise between the ridge and L_1_ penalties. While the nnlasso algorithm also starts with the smallest value of the regularization parameter, λ_*max*_, at which only the intercept is non-zero, it then uses a sequence of *k* λ values that are evenly spaced on a linear scale, and then uses a multiplicative iterative-Armijo algorithm for estimating model parameters under a non-negative constraint at each value of λ. While various penalized Poisson models can be fit to high-dimensional data, the focus of this paper is to extend the generalized monotone incremental forward stagewise (GMIFS) method to the negative binomial regression setting and demonstrate its effectiveness analyzing over-dispersed count outcome data.

### Proposed NB GMIFS

The proven GMIFS technique from our previous studies [58, 60–62] will be extended to a new setting, the NB model. The NB model is derived from a Poisson-gamma mixture distribution [38], such that the variance is *μ* + *μ*^2^/*ϕ*. Unlike the Poisson model, *ϕ* must be estimated. Most often the parameter *ϕ* is expressed as its inverse, *α* = 1/*ϕ*, such that *α* is the heterogeneity parameter that directly models the amount of extra-dispersion in the data. Our proposed NB GMIFS method includes approaches for initializing the intercept, coefficients for the unpenalized subset of predictors, and heterogeneity parameter *α*; methods for updating these estimates after each iterative update of the penalized subset of predictor variables; derivatives for identifying which covariate to update; and convergence criteria. The NB probability mass function is given by,

$$\begin{array}{c} f\left(y;\mu ,\alpha \right)=\frac{\Gamma \left(y_{i}+1/\alpha \right)}{\Gamma \left(y_{i}+1\right)\Gamma \left(1/\alpha \right)}{\left(\frac{1}{1+\alpha \mu_{i}}\right)}^{1/\alpha }{\left(1-\frac{1}{1+\alpha \mu_{i}}\right)}^{y_{i}} \end{array}$$ (5)

so the likelihood is

$$\begin{array}{c} L\left(\mu ;y,\alpha \right)=\prod_{i=1}^{N}\frac{\Gamma \left(y_{i}+1/\alpha \right)}{\Gamma \left(y_{i}+1\right)\Gamma \left(1/\alpha \right)}{\left(\frac{1}{1+\alpha \mu_{i}}\right)}^{1/\alpha }{\left(1-\frac{1}{1+\alpha \mu_{i}}\right)}^{y_{i}} \end{array}$$ (6)

which can be re-arranged as

$$\begin{array}{c} \begin{array}{cc} L(\mu ;y,\alpha )= & \prod_{i=1}^{N}\exp(\log\Gamma \left(y_{i}+1/\alpha \right)-\log\Gamma \left(y_{i}+1\right)-\log\Gamma \left(1/\alpha \right)+\\ & 1/\alpha \log\left(\frac{1}{1+\alpha \mu_{i}}\right)+y_{i}\log\left(1-\frac{1}{1+\alpha \mu_{i}}\right)) \end{array} \end{array}$$ (7)

Similar to Poisson regression, the log link function is used and when the outcome is a rate with the offset term *c*_*i*_ such that $E\left(y_{i}/c_{i}\right)=\mu_{i}$ or $E\left(y_{i}\right)=c_{i}\mu_{i}$. Therefore, the likelihood is

$$\begin{array}{c} \begin{array}{cc} L(\mu ;y,\alpha )= & \prod_{i=1}^{N}\exp(\log\Gamma \left(y_{i}+1/\alpha \right)-\log\Gamma \left(y_{i}+1\right)-\log\Gamma \left(1/\alpha \right)+\\ & 1/\alpha \log\left(\frac{1}{1+\alpha \mu_{i}c_{i}}\right)+y_{i}\log\left(1-\frac{1}{1+\alpha \mu_{i}c_{i}}\right)) \end{array} \end{array}$$ (8)

and the corresponding log-likelihood is

$$\begin{array}{c} \begin{array}{cc} \ell (\mu ;y,\alpha )= & \sum_{i=1}^{N}(\log\Gamma \left(y_{i}+1/\alpha \right)-\log\Gamma \left(y_{i}+1\right)-\log\Gamma \left(1/\alpha \right)+\\ & 1/\alpha \log\left(\frac{1}{1+\alpha \mu_{i}c_{i}}\right)+y_{i}\log\left(1-\frac{1}{1+\alpha \mu_{i}c_{i}}\right)) \end{array}. \end{array}$$ (9)

Given $E\left(y_{i}/c_{i}\right)=\mu_{i}=\exp\left(\gamma_{0}+x_{i}^{⊤}\beta \right)$ such that $E\left(y_{i}\right)=c_{i}\exp\left(\gamma_{0}+x_{i}^{⊤}\beta \right)=\exp\left(\gamma_{0}+x_{i}^{⊤}\beta +\log\left(c_{i}\right)\right)$, the log-likelihood with covariates reflected is

$$\begin{array}{c} \begin{array}{cc} \ell (\beta ;y,\alpha )= & \sum_{i=1}^{N}(\log\Gamma \left(y_{i}+1/\alpha \right)-\log\Gamma \left(y_{i}+1\right)-\log\Gamma \left(1/\alpha \right)\\ & +1/\alpha \log(\frac{1}{1+\alpha \exp(\gamma_{0}+x_{i}^{⊤}\beta +\log\left(c_{i}\right))})\\ & +y_{i}\log\left(1-\frac{1}{1+\alpha \exp(\gamma_{0}+x_{i}^{⊤}\beta +\log\left(c_{i}\right))}\right)) \end{array} \end{array}$$ (10)

The heterogeneity parameter in the NB model that accounts for the extra-dispersion can be selected *a priori* or estimated outside of the GLM framework then treated as a constant. Most NB modeling software employs iteratively re-weighted least squares, where *α* is estimated and its estimand is inserted into the equation as a constant and maximum likelihood estimation is applied. We examined the performance of three different methods (maximum likelihood, equating the deviance to the residual degrees of freedom, and Hilbe’s method of moments), for estimating the heterogeneity parameter using a small simulation study and found that Hilbe’s method of moments estimator performed well. Therefore in our proposed GMIFS NB model, we estimate *α* using Hilbe’s method of moments estimator prior to the iterative procedure [38]. Similar to our GMIFS Poisson approach, we partition the design matrix, **x**, in Eq 10 into two parts, **x**_*j*_ and **x**_*k*_, where *j* = 1, …, *J* is the set of unpenalized predictors, *k* = 1, …, *K* is the set of penalized predictors and *J* + *K* = *P*. The unpenalized predictors are those that we wish to force into the model, such as gender, age and smoking status which researchers consider important predictors of MN frequency [37] and their values are in the **x**_*ij*_ design matrix for subject *i*. The thousands of features from a high-throughput genomic experiment are the penalized predictors for which we seek a parsimonious model and their values are in the **x**_*ik*_ design matrix for subject *i*. The parameter vectors ***γ*** and ***β*** correspond to the unpenalized subset and penalized subset of predictors, respectively.

The algorithm proceeds in an iterative fashion and updates one of the penalized covariates by a small incremental amount at each step. To determine which penalized covariate is to be updated at each step the gradient of the log-likelihood is used. Thus we need to calculate the first derivative of the log-likelihood corresponding to each penalized predictor. The log-likelihood written in terms of *γ*_0_, ***β*** and ***γ*** is

$$\begin{array}{c} \begin{array}{cc} \ell (\beta ;y,\alpha )= & \sum_{i=1}^{N}(\log\Gamma \left(y_{i}+1/\alpha \right)-\log\Gamma \left(y_{i}+1\right)-\log\Gamma \left(1/\alpha \right)\\ & +1/\alpha \log(\frac{1}{1+\alpha \exp(\gamma_{0}+x_{ij}^{⊤}\gamma +x_{ik}^{⊤}\beta +\log\left(c_{i}\right))})\\ & +y_{i}\log\left(1-\frac{1}{1+\alpha \exp(\gamma_{0}+x_{ij}^{⊤}\gamma +x_{ik}^{⊤}\beta +\log\left(c_{i}\right))}\right)) \end{array} \end{array}$$ (11)

and the first derivative with respect to *β* written in terms of *γ*_0_, ***β*** and ***γ*** in matrix notation is

$$\begin{array}{c} \frac{\partial \ell }{\partial \beta }=x_{p_{k}}^{⊤}\left(y-\exp\left(\gamma_{0}+\log\left(c_{i}\right)+x_{j}^{⊤}\gamma +x_{k}^{⊤}\beta \right)\right)/\left(1+\alpha \exp\left(\gamma_{0}+\log\left(c_{i}\right)+x_{j}^{⊤}\gamma +x_{k}^{⊤}\beta \right)\right). \end{array}$$ (12)

Once we know which covariate to update, we need to determine the sign (+ or −) of the update. Rather than calculating the second derivative, an expanded covariate space can be used to get the direction of the update [55]. Using the previous notation for the unpenalized (**x**_*j*_) and penalized (**x**_*k*_) variables, the expanded covariate space is $\tilde{x}=\left[x_{j}:x_{k}:-x_{k}\right]$ where [**x**_*k*_: −**x**_*k*_] have been standardized. For the penalized predictors in the [**x**_*k*_: −**x**_*k*_] component, let *β*_1_, …, *β*_*K*_ be the positive coefficient estimates and *β*_*K*+1_, …, *β*_2*K*_ be the coefficient estimates of the negative version of **x**_*k*_. Our NB GMIFS algorithm using the expanded covariate set is

1. Initialize the components of ${\hat{\beta }}^{\left(s\right)}=0$ at step *s* = 0 and initialize *α* using Hilbe’s method of moments.
2. Estimate the intercept *γ*_0_ and the unpenalized coefficients *γ*_*j*_ where *j* = 1, …, *J* using a maximization algorithm of the log-likelihood.
3. Considering $\hat{\alpha }$, ${\hat{\gamma }}_{0}$ and $\hat{\gamma }$ fixed, find the predictor **x**_*m*_ where $m=\underset{2K}{\text{argmin}}(-\frac{\partial \ell }{\partial \beta_{k}})$ at the current estimate $\hat{\beta }={\hat{\beta }}^{\left(s\right)}$.
4. Update the corresponding coefficient ${\hat{\beta }}_{m}^{\left(s+1\right)}\leftarrow {\hat{\beta }}_{m}^{\left(s\right)}+\epsilon$ to yield a new vector of parameter estimates.
5. Update *γ*_0_ and the unpenalized coefficients, *γ*_*j*_, by maximum likelihood considering the ${\hat{\beta }}^{s+1}$ from step 4 as fixed.
6. Re-estimate *α* using Hilbe’s method of moments method.
7. Repeat steps 2-6 until the difference between successive log-likelihoods is less than a pre-specified tolerance, *τ*.
8. The final coefficient estimates are obtained by subtracting the pairs, *β*_1_ − *β*_*K*+1_, …, *β*_*K*_ − *β*_2*K*_, to yield $\hat{\beta }$.

Hastie et al (2007) [55] did not specify a stopping criteria but recommended to repeat the steps many times. Our criteria was to stop the iterative process when the difference between two successive log-likelihoods is smaller than a pre-specified tolerance *τ* or when the number of non-zero coefficient estimates exceeds *N* − 1, and we set the defaults to *ϵ* = 0.001 and *τ* = 0.00001. Further, recall that for the linear regression the intercept *γ*_0_ is commonly omitted because the response is centered. However, for count models it is inappropriate to center the outcome as that would result in negative counts. Therefore, the intercept must be included in the model without penalization, in addition to any covariates in the unpenalized subset. Once the iterative process has completed, the output includes a solution path for each coefficient. A ‘final’ model can then be selected from the resulting solution path based on predetermined desired criteria, such as the model attaining the minimum AIC, minimum BIC, or minimum cross-validated error.

### Simulation studies

Simulation studies were performed to compare our negative binomial GMIFS model to existing penalized count response models including glmpath [68], glmnet [54], and nnlasso [64]. First, we randomly generated *P* predictor variables for observations *i* = 1, …, *N* from a standard normal distribution. Thereafter five of the *P* variables were selected to be associated with the discrete response and their coefficients were set to *β* = ±log(*δ*). We also assigned an intercept to take the value of *γ*_0_ = 0.5 and we assigned a heterogeneity parameter *α*. We then calculated the mean response per observation as $\mu_{i}=\exp\left(\gamma_{0}+\sum_{k=1}^{5}\beta_{k}x_{ik}\right)$. The discrete response was then generated as *Y*_*i*_ ∼ Negative Binomial(*μ*_*i*_, *α*). Once the discrete response was generated, we fit the following models: our negative binomial GMIFS model, a glmpath model with family = poisson, a glmnet model with family = “poisson”, and a nnlasso model with family = “poisson”. These methods maximize the log-likelihood with the additional penalty term λ placed on the regression coefficients, $\ell \left(\beta ;y,\alpha \right)-\lambda \sum_{p=1}^{P}\left|\beta_{p}\right|$. To ensure a fair comparison across the four modeling methods, we extracted the GMIFS model that attained the minimum AIC and minimum BIC, summed the absolute values of the estimated regression coefficients, then identified the step at which glmpath, glmnet, and nnlasso first attained a sum of the absolute value of the regression coefficients at the GMIFS level. This procedure was repeated *r* = 200 times. Simulations were performed using *N* = 100, *P* = 500, *α* = 0.3 and 0.5, and *δ* = 1.5 and *δ* = 1.75. The four methods were compared with respect to the number of true predictors that had a non-zero coefficient estimate; the number of false predictors that had a non-zero coefficient estimate; and prediction error.

A larger simulation study consisting of (*P* = 5, 000) correlated rather than independent features and *N* = 50 observations was also performed to mimic the structure of our application dataset. In the MoBa dataset, heterogeneity parameter estimate for the BIC-selected model was ${\hat{\alpha }}_{BIC}=0.337$ and the six parameter estimates from the unpenalized model ranged from [-0.988, 1.122]. Therefore we set *α* = 0.35, the intercept to *γ*_0_ = 0.5, and conservatively set the parameter values of the true covariates to *β* = ±0.693, which corresponds to *β* = ±log(2). We also estimated the correlations between genes included in the final model and all remaining genes in the dataset. This distribution is approximately normally distributed with a mean of 0.005 and a standard deviation of 0.28. Therefore, we developed a block diagonal correlation matrix with 40 features in each block and 125 blocks in total, to yield a 5000 × 5000 correlation matrix. First, the lower triangle of each 40 × 40 block of the correlation matrix was filled by generating random variates from a N(0,0.28) distribution. Second, the upper triangle was completed by enforcing the matrix to be symmetric. Third, the diagonal elements were taken to be 1. Thereafter, one feature from five different blocks was selected to represent the true covariates. The mean response per observation was taken to be $\mu_{i}=\exp\left(\gamma_{0}+\sum_{k=1}^{5}\beta_{k}x_{ik}\right)$ and the discrete response was then generated as *Y*_*i*_ ∼ Negative Binomial(*μ*_*i*_, *α*). Once the discrete response was generated, we fit our negative binomial GMIFS model and the glmnet model with family = “poisson”. This procedure was repeated *r* = 100 times. Similar to [54], we omitted comparison to the glmpath algorithm because the algorithm does not scale well to the large size of this simulated dataset. Also, we omitted the comparison to nnlasso because, as demonstrated in the small simulation study, its non-negative constraint on the parameters results in poor performance when parameters can take both positive and negative values. The two methods were compared with respect to the number of true predictors that had a non-zero coefficient estimate; the number of false predictors that had a non-zero coefficient estimate; and prediction error. All simulations were performed on the Ohio Supercomputer Center cluster owens [69].

### Norwegian Mother and Child Cohort Study

In the 1990s the Norwegian Mother and Child Cohort Study (MoBa) was designed collaboratively by researchers at the Medical Birth Registry of Norway (MBRN) and by researchers at the National Institute of Public Health [70]. Pregnant women who attended routine ultrasounds in Norway were recruited from 1999 to 2005 from 52 hospitals and maternity units. There was no exclusion criteria, and women who were pregnant more than once in the time period could participate multiple times. The pregnancy was defined as the unit of observation of the study. For 200 neonates, umbilical cord blood samples were collected immediately after birth. After quality control and other exclusions, 111 samples were hybridized to Agilent 4x44k human oligonucleotide microarrays to measure gene expression. Sample processing, image analysis, normalization, background correction, and filtering for the gene expression data are described in [71]. For an even smaller subset (*N* = 29), MN data were collected and scored using the procedure previously described [72]. Data were downloaded from Gene Expression Omnibus (GSE31836).

## Results

### Simulation studies

**Fig 3:**
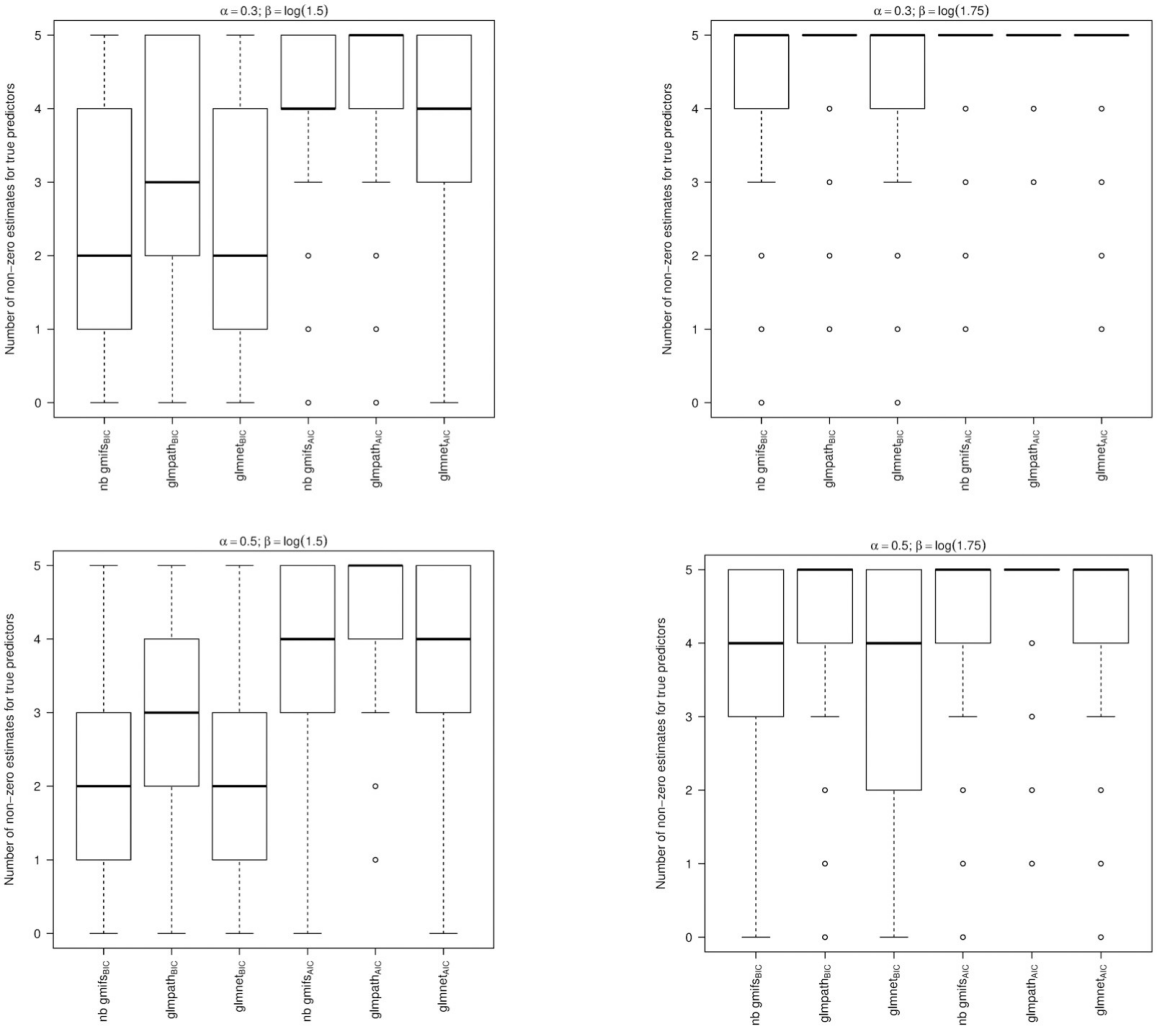
For each modeling method, number of true predictors that had a non-zero coefficient estimate (Oracle = 5). A: *α* = 0.3; *β* = ±log(1.5). B: *α* = 0.3; *β* = ±log(1.75). C: *α* = 0.5; *β* = ±log(1.5). D: *α* = 0.5; *β* = ±log(1.75).

**Fig 4:**
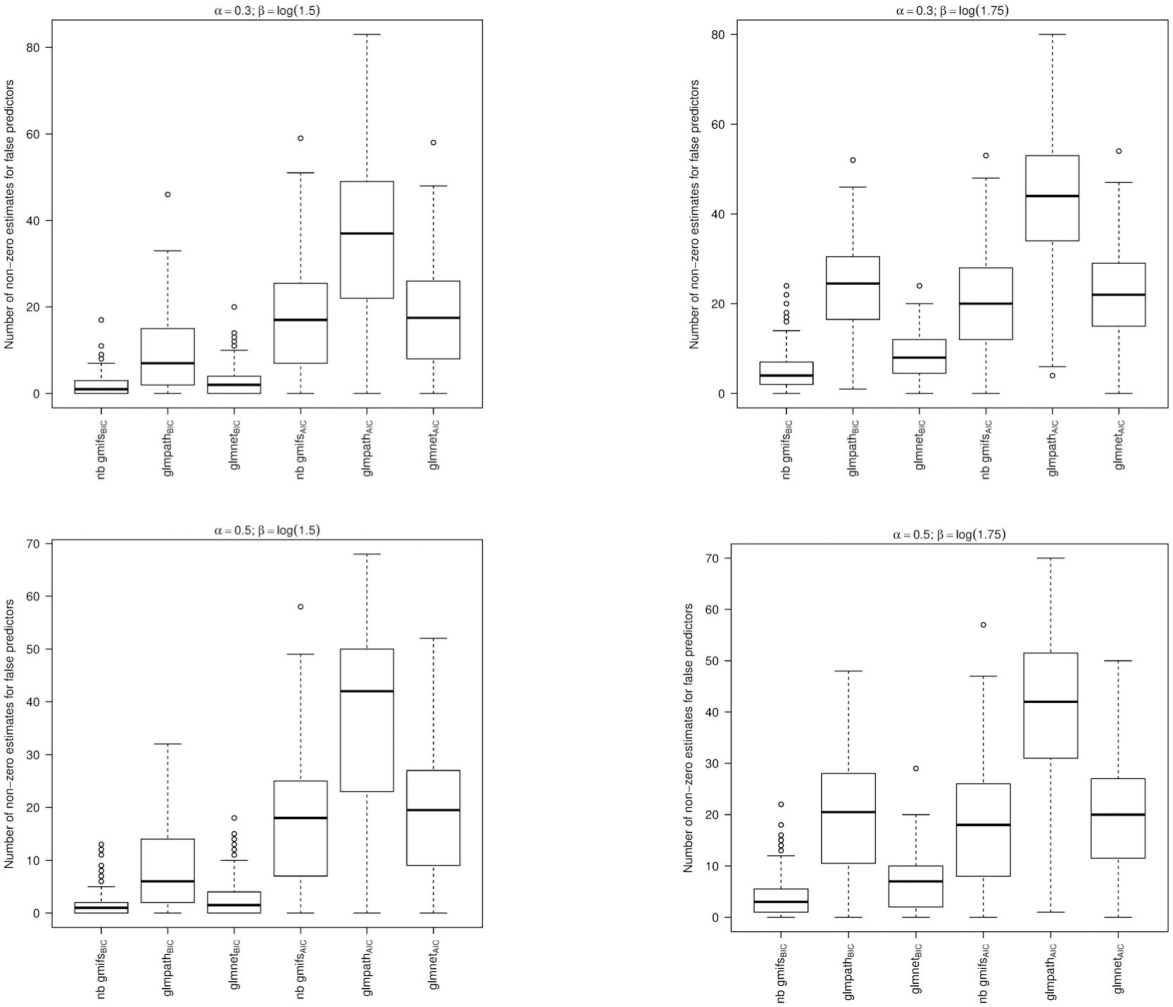
For each modeling method, number of false predictors that had a non-zero coefficient estimate (Oracle = 495). A: *α* = 0.3; *β* = ±log(1.5). B: *α* = 0.3; *β* = ±log(1.75). C: *α* = 0.5; *β* = ±log(1.5). D: *α* = 0.5; *β* = ±log(1.75).

**Fig 5:**
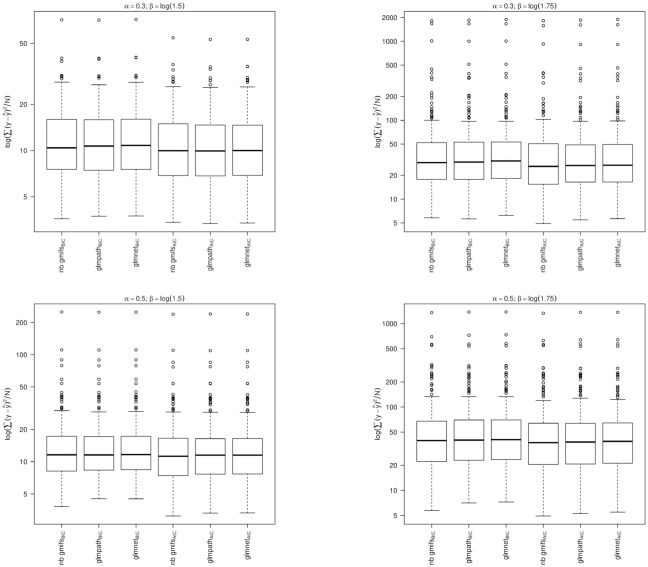
Prediction error for each modeling method and selection criteria. A: *α* = 0.3; *β* = ±log(1.5). B: *α* = 0.3; *β* = ±log(1.75). C: *α* = 0.5; *β* = ±log(1.5). D: *α* = 0.5; *β* = ±log(1.75).

From the simulation studies we observed that the nnlasso method had poor performance, with the maximum number of false predictors included in the BIC-selected models extending to 292, 296, 298, and 291 when *α* = 0.3 and *β* = ±log(1.5) or ±log(1.75) and for *α* = 0.5 and *β* = ±log(1.5) or ±log(1.75), respectively, which may be due to its non-negativity constraint on the parameter estimates. Therefore the nnlasso results were omitted from the boxplots because their inclusion obfuscated the results for the other methods. When examining the boxplots of the simulation results, we observed that our NB GMIFS method performed well with respect to identifying true predictors (Fig 3) while minimizing the number of false predictors included in the model (Fig 4), particularly as *α* and *β* increased. We also observed that glmnet performed comparable to our method, though our NB GMIFS method performed better than glmnet when identifying true predictors for AIC selected models when *α* = 0.3 and *β* = ±log(1.5) and for BIC selected models when *α* = 0.5 and *β* = ±log(1.75) (Fig 3). Additionally, our NB GMIFS method identified fewer false predictors, especially for the two *β* = ±log(1.75) scenarios (Fig 4). While glmpath performed well at selecting the true predictors, it over-selected false predictors (Fig 4). Because our negative binomial GMIFS model had good performance with respect to prediction error (Fig 5) and minimized the number of false predictors included while doing well at selecting the true predictors, it is preferred for modeling an outcome that is over-dispersed.

**Fig 6:**
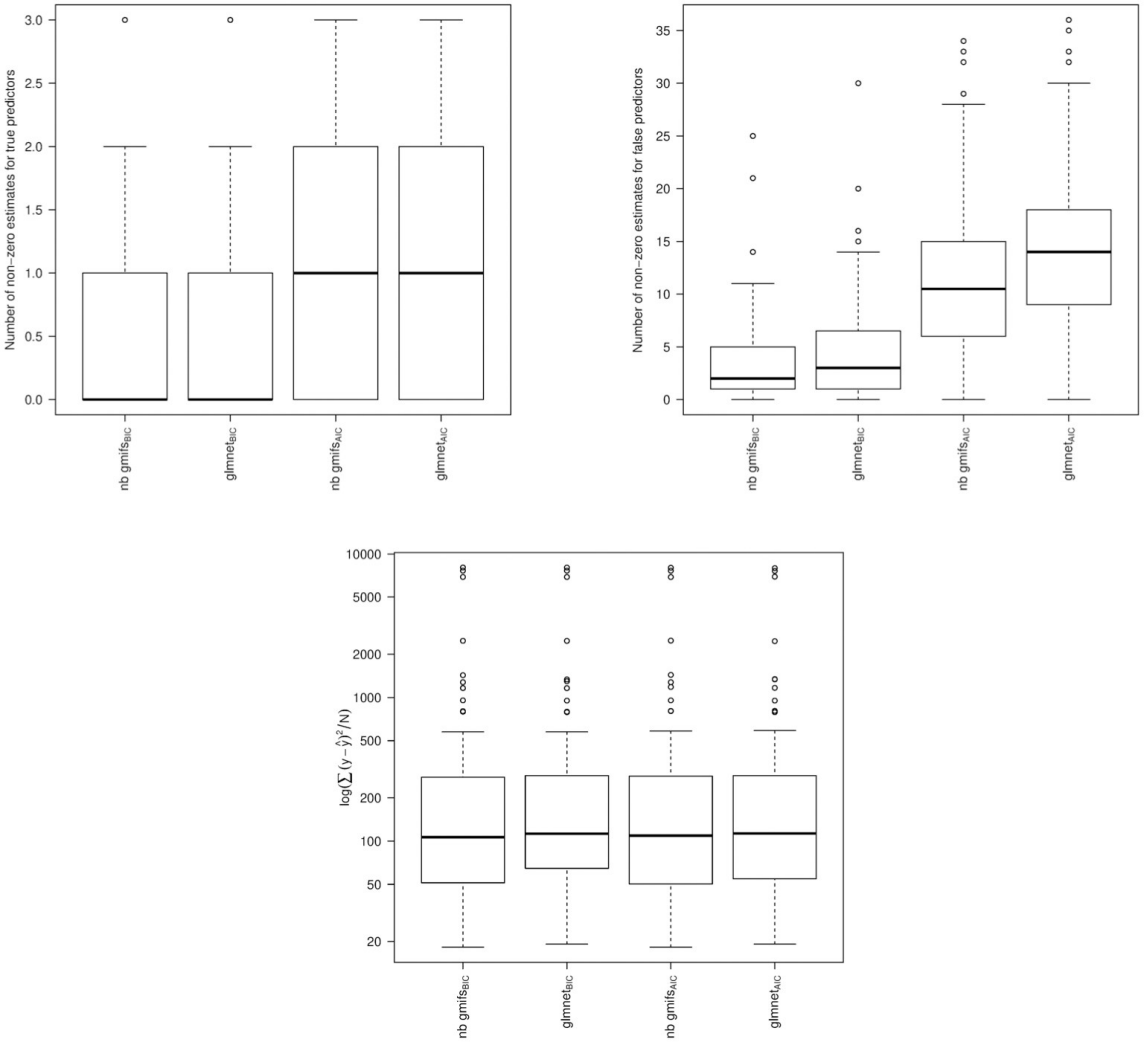
Results from large simulation study of *P* = 5, 000 correlated predictors and *N* = 50 observations. A: Number of true predictors that had a non-zero coefficient estimate (Oracle = 5). B: Number of false predictors that had a non-zero coefficient estimate (Oracle = 4,995). C: Prediction error for each modeling method and selection criteria.

When examining the results from the larger simulation that consisted of *P* = 5, 000 correlated covariates for *N* = 50 subjects, we observed similar performance between our NB GMIFS method and glmnet with respect to identifying true predictors (Fig 6). However, our NB GMIFS method included fewer false predictors than glmnet (Wilcoxon signed rank test *P* < 0.0001 and *P* < 0.0001 for the AIC- and BIC-selected models, respectively) (Fig 6).

### Norwegian Mother and Child Cohort Study

Before statistical analysis, a Boundary Likelihood Ratio test was performed to determine whether a Poisson or negative binomial model would be more appropriate given the MoBa data [38]. The alternative hypothesis of *α* ≠ 0 was tested against a null hypothesis of *α* = 0. The chi-square test yielded $\chi_{1}^{2}=59.8$ which corresponds to a p-value of < 0.0001. Therefore, we rejected the null hypothesis that *α* = 0 and concluded a negative binomial model is more appropriate given the data. When applying the score test to test the null hypothesis of no over-dispersion against the alternative hypothesis that over-dispersion is present, *P* = 0.011. When testing for over-dispersion using the Lagrange multiplier test, *P* < 0.0001; therefore, all tests indicated over-dispersion is present such that the NB model is preferred to the Poisson. Because the feature set is high-dimensional gene expression data, we used our GMIFS NB model to fit a multivariable model to predict MN frequency. We further filtered the gene expression dataset to include genes that had no missing values, leaving 8,497 genes for statistical modeling. Because the performance of glmnet and glmpath was somewhat comparable to our GMIFS method in the simulation studies, with nnlasso demonstrating poor performance likely owing to its non-negativity constraint, we applied glmnet and glmpath to the MoBa data as comparative methods.

**Fig 7:**
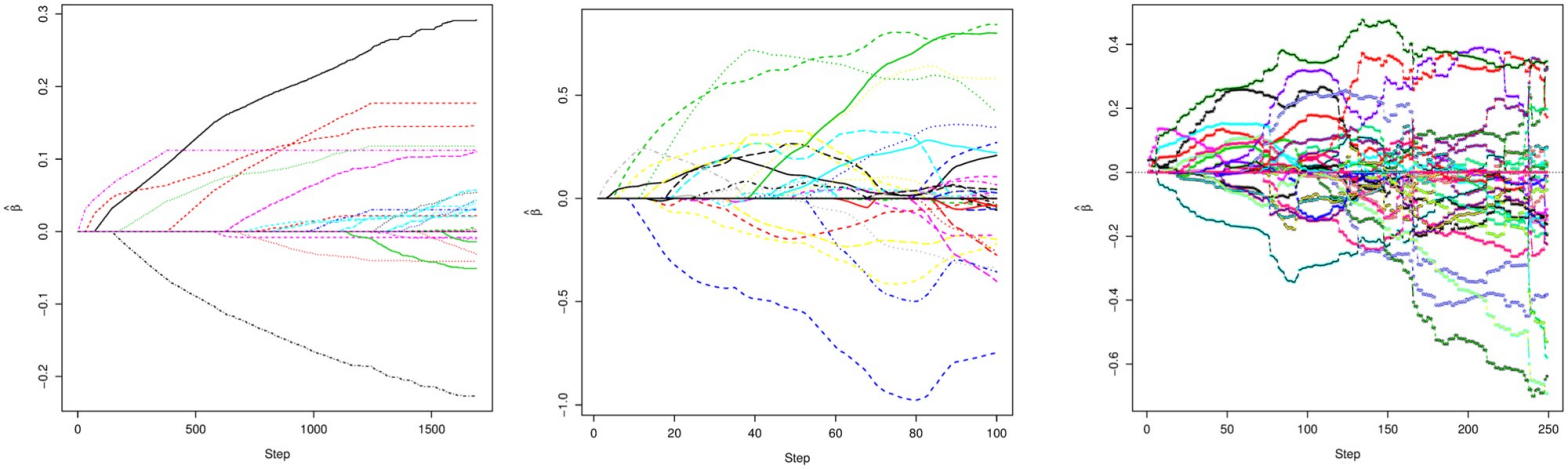
Coefficient paths for each modeling method for the MoBa dataset. A: Coefficient path from NB GMIFS. B: Coefficient path from glmnet where the x-axis indicates the sequence number of λ. C: Coefficient path from glmpath.

Though maternal age, gestational age, and maternal smoking status would ordinarily be of interest to include a unpenalized predictors, those data were not available so the only unpenalized predictor included in our model was neonate gender. The gene expression data were included in the model as penalized predictors. There were 13 genes with non-zero coefficient estimates in the AIC selected NB GMIFS model and six in the BIC selected NB GMIFS model (Table 1). The BIC attained a minimum at step 580 while the AIC attained its minimum at step 1102. The AIC selected glmpath Poisson model included 23 genes while the BIC selected glmpath Poisson model included 17 genes, so similar to our simulation studies, glmpath seems to overfit. Nine of the genes from the AIC selected NB GMIFS and glmpath Poisson models overlapped. Again, because glmnet does not include functions or relevant output for estimating AIC and BIC, we extracted the GMIFS models that attained the minimum AIC and minimum BIC, summed the absolute values of the estimated regression coefficients, then identified the step at which glmnet first attained a sum of the absolute value of the regression coefficients at the GMIFS level. The AIC-like glmnet Poisson model included nine genes while the BIC selected glmnet Poisson model included five genes; seven of the genes from the AIC selected NB GMIFS and glmnet Poisson models overlapped. As suspected, the monotone solution paths for NB GMIFS are much smoother than those produced by glmnet, likely due to the correlated nature of the covariates (Fig 7). While the mean prediction error for the AIC- and BIC-selected models from our NB GMIFS algorithm were 2.21 and 3.23, respectively, the mean prediction errors were 3.86 and 3.30 for glmnet. As expected, the generalization error was larger as the N-fold cross-validation estimates of mean prediction error were 5.40 and 5.18 for the AIC- and BIC-selected NB GMIFS models, respectively, and 5.42 and 4.99 for the AIC and BIC glmnet models, respectively.

**Table 1:** Genes associated with MN frequency in the AIC and BIC selected NB GMIFS models.

Accession ID	Gene Symbol	Gene name	NB GMIFS AIC	NB GMIFS BIC	glmpath AIC	glmpath BIC	glmnet AIC	glmnet BIC
A_23_P100196	USP10	ubiquitin specific peptidase 10	X	X	X	X	X	X
A_23_P103824	THC2249577						X	
A_23_P133424	SKP1		X					
A_23_P138967	SDHD	succinate dehydrogenase complex	X		X			
A_23_P209394	CFLAR	CASP8 and FADD-like apoptosis regulator	X				X	
A_23_P42331	HMGA1	high mobility group AT-hook 1	X		X			
A_23_P79911	PSMF1	proteasome (prosome, macropain) inhibitor subunit 1 (PI31)					X	
A_24_P19410	CBX7	chromobox homolog 7	X	X	X	X	X	
A_24_P214858	TREML2	triggering receptor expressed on myeloid cells-like 2	X		X			
A_24_P2463	WHSC1	NSD2, Wolf-Hirschhorn syndrome candidate 1	X	X	X	X	X	X
A_24_P333019	RNF24	ring finger protein 24	X					
A_24_P397584	TBCC	tubulin folding cofactor C	X		X			
A_24_P398064	KIAA0258	RGP1 homolog, RAB6A GEF complex partner 1	X	X	X	X	X	X
A_32_P156549	C1ORF144		X	X			X	X
A_32_P18547	C21ORF57	chromosome 21 open reading frame 57	X	X	X	X	X	X

Interestingly, genes included in all models predicting MN frequency have been previously linked to relevant disease processes and cancer. For example, USP10 has been previously found to be involved in autophagy [73] and DNA damage response of cells [74]. CBX7 has been associated with thyroid [75] and endometrial cancer [76]. WHSC1, which is a synonym for NSD2, is involved in morphogenesis of anatomic structure [77] and associated with hematologic malignancies [77–79] and hepatocellular carcinoma [80]. KIAA0258 is a synonym RGP1. A mutation in RGP1 has been associated with adenocarcinoma of the large intestine [81]. C21ORF57 is a synonym for YBEY which according to COSM1031614, mutations in this gene in two TCGA samples have been associated with endometrioid carcinoma.

## Discussion

The simulation studies established that when the underlying data follow a negative binomial distribution (that is, the outcome is over-dispersed) the negative binomial GMIFS model outperforms penalized Poisson models with respect to including fewer false predictors. It also includes a substantial number of true predictors, particularly when the strength of association between the outcome and the predictor variable increases. Often it is of interest to account for the denominator, e.g., the number of binucleated cells scored, in the model, particularly if it varies by subject. This is usually accommodated by generalized linear modeling software by specifying the denominator to be an offset. While glmpath and glmnet include a parameter to the function call that allows for inclusion on an offset, both suffered from convergence issues when including an offset term. Also, the current implementation of nnlasso does not permit inclusion of an offset, which we consider to be a limitation of that package. Inclusion of an offset is not problematic for our GMIFS procedure given its incremental nature. Therefore our negative binomial GMIFS model offers several advantages.

Using various goodness-of-fit tests, it was determined that the micronuclei frequencies observed in the MoBa study more closely followed a negative binomial rather than a Poisson distribution. That finding is consistent with what has previously been reported [37]. When our NB GMIFS model was applied to the MoBa dataset, interesting genes that have previously been associated with cancer or relevant processes were identified, though genes that are truly associated with MN frequency in the MoBa study are unknown. One limitation is that when estimating Pearson’s correlation, $\hat{\rho }$, between all remaining genes and the 13 genes included in the AIC-selected model, 316 were significantly correlated at an FDR< 0.05. Although the distribution of the correlation estimates appeared Gaussian and centered near zero ($\bar{\rho }=0.005$ and ${\hat{\sigma }}_{\rho }=0.28$), among the 316 significant genes, the absolute value of the estimated correlations ranged from 0.718 to 0.889. Therefore, it may be of scientific interest to explore the biological functions of these 316 genes because penalized methods tend to omit variables from the model if good proxies are already included.

## Conclusion

Though the proposed NB GMIFS method was conceived of by considering high-throughput genomic applications, it is broadly applicable to a variety of health, social, and behavioral research fields, which commonly collect responses on an discrete scale. For example, our method is broadly applicable for modeling other discrete responses in high-dimensions, using the large number of predictors from an Electronic Medical Record system, for outcomes including but not limited to: length of hospital stay, number of drinks per day, and number of positive lymph nodes. An essential aspect for the dissemination of statistical methods is the development of software for the scientific user community. Therefore, our countgmifs package for the widely used R programming environment is available for download from the Comprehensive R Archive Network for others to model a discrete response, using either our Poisson of NB GMIFS method, when the covariate space is high-dimensional.
